# Fine-scale behavioural differences distinguish resource use by ecomorphs in a closed ecosystem

**DOI:** 10.1038/srep24369

**Published:** 2016-04-21

**Authors:** Kate L. Hawley, Carolyn M. Rosten, Guttorm Christensen, Martyn C. Lucas

**Affiliations:** 1 Norwegian Institute for Water Research (NIVA), Gaustadalléen 21, 0349 Oslo, Norway; 2 School of Biological and Biomedical Sciences, Durham University, Science Laboratories, South Road, Durham, DH1 3LE, UK; 3 Norwegian Institute for Nature Research (NINA), Høgskoleringen 9, 7034 Trondheim, Norway; 4 Akvaplan-NIVA, Fram Centre, 9296 Tromsø, Norway

## Abstract

Temporal differences in habitat use and foraging specialisms between ecomorphs represent aspects of behavioural phenotype that are poorly understood with regard to the origin and maintenance of ecological diversity. We tested the role of behaviour in resource use divergence of two Arctic charr (*Salvelinus alpinus*) phenotypes, a slim, putatively pelagic-dwelling morph and a robust, putatively littoral-dwelling generalist morph, over an annual cycle, using biotelemetry and stable isotopes. Pelagic morph charr exhibited significantly greater δC^13^ depletion, concordant with increased zooplanktivory, than for the Littoral morph. Although three-dimensional space-use of the morphs strongly overlapped, on average, the Littoral morph used that habitat 19.3% more than the Pelagic morph. Pelagic morph fish were significantly more active, further from the lake bed and at greater depth than Littoral fish (annual means respectively, Pelagic, 0.069BLs^−1^, 8.21 m and 14.11 m; Littoral, 0.047BLs^−1^, 5.87 m and 10.47 m). Patterns of habitat use differed between ecomorphs at key times, such as during autumn and at ice break, likely related to spawning and resumption of intensive foraging respectively. Extensive space-use overlap, but fine-scale differences in habitat use between charr ecomorphs, suggests the importance of competition for generating and maintaining polymorphism, and its potential for promoting reproductive isolation and evolution in sympatry.

Resource polymorphism occurs when discrete intraspecific phenotypes (ecomorphs, *sensu*^1^) exhibit contrasting niche-based variation due to differential use of environments and their associated resources^2^. Ecological processes, especially intraspecific competition, combined with natural selection, has been suggested to promote polymorphism^1^. Polymorphism may promote reproductive isolation of sympatric phenotypes if the traits associated with differential resource use influence mate choice directly^3^,^4^. Differences in local resource use, both spatially and/or temporally, by phenotypes may also result in sympatric isolation and the subsequent divergence of populations^3^,^4^. Morphological diversification is often complex and while many taxa exhibit phenotypic plasticity, particularly during key developmental stages in response to the environment^5^,^6^, occurrence of distinct ecomorphs commonly also reflects underlying genetic divergence^3^,^7^,^8^,^9^,^10^. Yet, over a diverse range of taxa, functional relationships of morphology, habitat use and diet are often linked to improved performance in instances of competition^7^,^9^,^11^, ecological opportunity^10^,^12^ and predation^13^,^14^. Resource use polymorphism has been proposed as a key factor in sympatric population divergence^1^,^15^. Divergence according to this process often varies continually, even if the endpoint is the development of discontinuity^2^, and has been decribed as a continuum, from individual variation in panmictic populations, to population wide phenotypic and genotypic polymorphisms with inceasing levels of reproductive isolation^16^,^17^. Thus, understanding how organisms respond to contemporary spatio-temporal environmental heterogeneity is a fundamental focus in ecology and evolutionary biology.

Some of the most remarkable examples of resource polymorphism are found in freshwater fishes; including cichlids^15^,^18^, whitefishes^10^,^19^, sticklebacks^20^ and charrs^21^,^22^; in these examples differences in relevant morphological and life history traits are observed, and the phenomenon of “speciation continua” according to the evolution of population divergence and eventual speciation, is seemingly common in these species^4^,^8^,^10^. Skúlason *et al.*^4^ suggest a model of incipient speciation whereby the first stage is the adaptive divergence of phenotype and the second stage is the evolution of reproductive isolation as a by-product of differential habitat use. Isolated lakes, including those of post-glacial origin, are often used as model systems to test such assumptions about the way in which divergence of populations may evolve^10^. Many of these post-glacial lakes are located in Arctic regions and are of relatively recent formation, characterised by low temperatures and strong seasonality in photoperiod and primary and secondary production. As a result of this, such lakes are of low biodiversity, frequently containing only a single fish species, commonly Arctic charr (*Salvelinus alpinus)*.

The Arctic charr exhibits high rates of resource polymorphism, and in many lakes containing charr, discrete pelagic, littoral and profundal benthic habitats are available and specialised phenotypes associated with these habitats can coexist^14^,^21^,^23^,^24^. The pelagic ecomorph usually specialises on zooplankton prey, whereas the littoral or deep-water benthic ecomorphs usually consume larger invertebrates associated with the shallow margins or profundal sediments^8^ respectively. In many instances, reproductive isolation among phenotypes has been observed and is likely to have occurred due to spatial or temporal segregation by habitat^4^,^8^,^23^,^25^. It is often unclear if Arctic charr ecomorphs occurring in sympatry are of allopatric or sympatric origin, and this likely varies between lake systems depending on their geography and colonisation history^26^. The degree of genetic divergence between ecomorphs varies among lake systems^26^, but is generally correlated with the degree of phenotypic divergence^14^, which usually occurs along ecological gradients that correlate with the number and availability of habitats and food resources in the lake^14^ and the number of fish species other than Arctic charr present^8^.

It is therefore evident that diversity in diet and habitat resource use is a key process in the development and maintenance of resource polymorphism, mediated partly through individual behaviour. Isolated lakes which contain charr as the sole fish species (mostly occurring in the Arctic) provide an ideal natural study system to evaluate the effects of seasonality and environmental heterogeneity on resource polymorphism, particularly the linkages between individual behaviour, morphology, diet and habitat use. However, limited year-round research has been conducted in Arctic and subarctic lakes due to the practical difficulties associated with this inhospitable environment^25^, with studies during the predominant winter season and the short but dynamic spring and autumn seasons often absent from the literature (but see for example^27^,^28^,^29^,^30^).

We deployed a novel autonomous method of telemetry, which allowed us to investigate to what extent spatio-temporal behaviour distinguishes conspecific phenotypes of the only fish species present in a closed (i.e. lake) system, over the course of a year. We hypothesised that behaviour most closely associated with prey choice (e.g. duration and timing of relevant habitat use) would show the strongest correlation with morphology, but we also hypothesised that, in small isolated lakes, like that studied, spatial segregation linked to trophic polymorphism, is incomplete and dynamic. Most studies that have examined habitat segregation directly by sampling e.g.^27^,^31^, or inferred it indirectly, by stable isotope analysis e.g.^32^ have exhibited limited temporal resolution, making it difficult to distinguish longer term patterns from shorter critical periods of dissimilarity. Telemetry provides unprecedented access into the habitat use patterns of wild animals, and yields information on habitat segregation and behaviour at a fine temporal resolution, as well as derivation of individual activity levels^33^. To test these hypotheses we quantified daily habitat use, mobility and seasonal activity over a whole year for individual charr, combined with morphometric analysis and stable isotope measurement of diet of the same individuals.

## Materials and Methods

### Study area

Lake Ellasjøen, with a maximum depth of 34 m, is located on Bear Island (74° 30’ N, 19° 00’ E), a remote, high Arctic island, equidistant between Spitsbergen (Svalbard archipelago) and mainland Norway. The littoral zone of Ellasjøen comprises a steep rocky margin and, except for occasional submerged mosses, no macro-vegetation is present. The cladoceran, *Daphnia longispina* and chironomids dominate the zooplankton and macrobenthos respectively^34^. Arctic charr is the only fish species in the lake, surveyed by Klemetsen *et al.*^34^ who suggested there were two morphs, characterised by differences in body size and growth rate. They observed that present day anadromy does not exist in Ellasjøen charr, as the outlet is too steep for fish to ascend.

**Figure 1:**
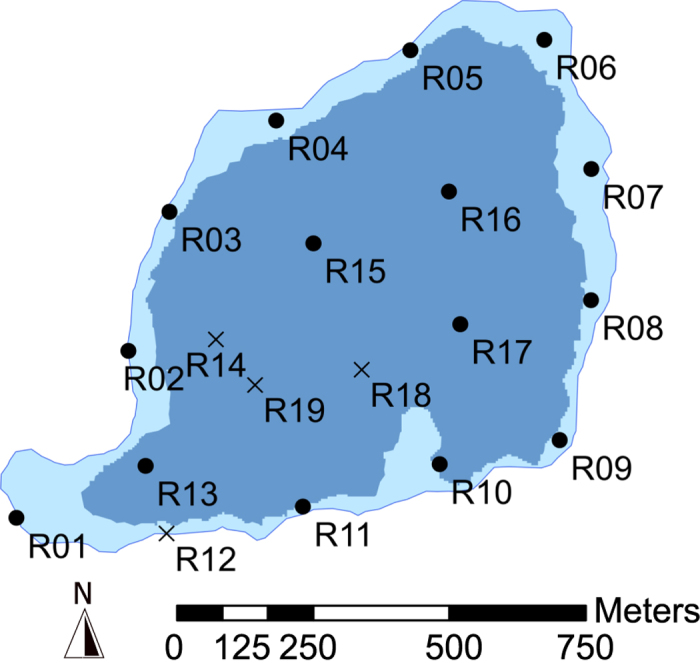
Deployment location of the 19 omnidirectional acoustic receivers (and co-located sync tags) of the acoustic positioning system, Lake Ellasjøen, Bear Island (28/8/2009–23/8/2010). Filled circles, represent those VR2Ws recovered, crosses those not recovered. The background map of Ellasjøen is shaded according to the depth of the lake; light blue 0–8 m (littoral zone), darker blue 9–34 m (offshore zone). Map was created using ESRI (Environmental Systems Resource Institute http://www.esri.com/software/arcgis) ArcMap software, version 10.0, 2010.

Three temperature loggers (Vemco: V13T-1L), set at 3, 25 and 31 m depths, recorded the water temperature of Ellasjøen over the study period (28/8/2009–23/8/2010). The lake showed negligible stratification over summer (Fig. S1) but an inverse temperature gradient occurred over winter, inferring the likely period of complete ice coverage (16/12/2009–24/5/2010, 158 days), with temperatures close to zero at 3 m between December and June. Polar night occurred between 8/11/2009–3/2/2010 (88 days) and polar day occurred between 31/4/2010–12/8/2010 (102 days), based upon times of sunrise and sunset (U.S. Naval Observatory website http://aa.usno.navy.mil/data). The surface area of Ellasjøen (0.71 km^2^) was divided, based on Secchi measurements into a littoral (0–8 m where total depth is ≤8 m), and offshore pelagic zone (the limnetic and profundal zones combined), forming 22.5% and 77.5% of Ellasjøen respectively (Fig. 1).

### Acoustic telemetry positioning system

This study used an autonomous acoustic telemetry array system, the VR2W Positioning System (VPS, Vemco, Halifax, Canada). This is a non-real-time, underwater acoustic positioning system, capable of providing near-continual spatial and temporal data of a tagged subject in an aquatic environment, provided the animal remains within the instrumented zone.

The system consists of receivers with omnidirectional hydrophones (VR2Ws) deployed in a grid formation, optimised for coverage of the study area. The receivers are downloaded and the data is extracted and processed. Transmitter (tag) location is calculated by hyperbolic positioning, using delays in time of arrival of acoustic signals between receivers in differing locations around the transmitter. The system generates a positional error equivalent or better than manual active tracking methods^35^, and was ideal for deployment in Ellasjøen as it enabled automated collection of fine-scale, individual positional data of charr *in-situ,* with deployment and retrieval only required during summer. Nineteen 69 KHz VR2W and ‘co-located’ synchronisation (sync) tag (V13–1L, code repeat rate 80 minutes) pairs were deployed underwater and assigned a station number (e.g. R01) (Fig. 1). The use of sync tags enabled millisecond synchronisation of surrounding receivers, and measurement of the effectiveness of each receiver recovered over its operation period (Fig. 1). The positional data derived were pre-treated (see^36^) in order to filter lower quality positional fixes due to suboptimal geometry between loggers, non-viable fixes, and disagreement in location depth at fix from array and fish tag depth data; a total of 15,416 (8.06%) of all VPS derived fish positions were excluded from further analysis.

### Fish sample and morphology

Fish were caught by rod and line from the lake shore to target littoral fish or by boat over the deepest area to target pelagic fish. Nordic multi-mesh (12 mesh sizes, 5–55 mm) bottom set gill nets (30 × 1.5 m) were also set in the deeper areas of the lake, to target benthic, profundal fish. They were set for a 24 hour period, 27–28 August 2009, lifted every 2–6 hours to minimise the period fish were in nets. The tags used on charr were 9-mm diameter acoustic transmitters with pressure sensors, capable of measuring depths up to 50 m (resolution 0.22 m) (V9P-6L, 2.9 g in air). Each tag had a code repeat rate quasi-randomly centred around 80 minutes. To minimise impacts on physiology and behaviour^33^, fish selected for tagging had a tag to body mass ratio less than 3.8% (average 1.1%) (Table S1). Fish were selected for tagging based upon differences in body form, colouration, eye size and head shape, characteristic of Littoral and Pelagic morphs (see below) as well as being undamaged in external appearance and movement. Dwarf-maturing fish were not tagged due to the small number caught and their small size (mean fork length (FL) 213 mm), relative to the smallest depth-sensing tags available. Twenty four fish were surgically tagged at the lake edge; the surgical procedure is described in Appendix S1. Fish handling and surgical procedures were carried out in accordance with approved guidelines and all experimental protocols were approved by the Norwegian national authority for animal research (Forsøksdyrutvalget, FDU).

All charr, including the 24 fish (Table S1) subsequently tagged, were sedated and nine morphometric measurements were taken (the procedure is described in Appendix S1). Head measurements were selected based on their relationship to prey acquisition and handling^21^. Body shape measurements were selected to differentiate swimming modes in different foraging habitats^37^. Photography was used as additional evidence of any intra-population variation, in terms of colouration and markings and each photographed fish was subsequently assigned to a morphology grouping. Four visually distinct groups were defined (examples are given in Fig. S2): 1) putative Littoral morph: large size, orange/red colouration, white fin edges at the time of capture; 2) putative Pelagic morph: smaller size, silver colouration, pointed snout, large eye; 3) Dwarf –maturing morph: small size, mixed sex (gametes released when gentle pressure was applied), ‘parr’ markings; 4) Other: small size, immature (no gametes released when gentle pressure was applied), silver/parr colouration. The fourth group, Other (*n* = 3) were not analysed further in this study as these fish were a juvenile, intermediate form and not an explicit phenotype.

### Stable isotope analysis

Analysis of nitrogen and carbon stable isotopes was used to indicate long-term dietary niche of individual charr. The distal 5 mm of the left pelvic fin of each individual was clipped during anaesthesia, air dried in the field and a subsample of these (*n* = 25, Table S1) were stable isotope analysed (SIA) for δ^13^C and δ^15^N. The samples were processed according to Grey *et al.*^38^. Isotope ratios are expressed conventionally in per mille (‰) relative to a secondary standard of known relation to Vienna PeeDee Belemnite or atmospheric nitrogen for δ^13^C and δ^15^N respectively.

### Home range estimation

A sample of 54 positions per fish, based upon Incremental Area Analysis, according to Hodder *et al.*^39^ was used to estimate monthly home range. Positions were categorised by time (dawn, dusk, day, night, polar day and polar night) and grouped into 3-hour periods. Positions were then selected randomly from each group representing the proportion of each time category each month, thus minimising auto-correlation and imitating the highly varied Arctic photoperiod. Kernel Analysis was selected as the most appropriate home range estimator^40^. The 95% probability distribution zone, K95, was used to estimate the outer range area, and K50 (50% probability distribution zone) to represent the core range area. As home range estimates extended beyond the lake edge (i.e. onto land), estimates of K95 and K50 were ‘clipped’ to the feasible boundary, Lake Ellasjøen. All analyses were conducted in Ranges 8 (Anatrack Ltd, 2008) and generated individual monthly home range and core range values per fish (255 values for all fish and months).

### Statistical analysis

To test for membership to assigned charr morphology groups, morphometric data were first transformed to correct for body length by calculating the residual variation of body shape, from linear regressions of each measured variable^41^. Discriminant analysis was applied to the residual values of each trait to test for group membership. Stepwise forward insertion of variables was used to minimise the sum of unexplained variance for all groups and to identify those traits which discriminate between the groups. The model calculated the probability of each individual being correctly assigned to the visually designated groupings, based on how close the morphometric values of the individual were to the mean values of the group being predicted.

To assess whether patterns of behaviour and habitat use differed across months and between morphs, we employed Generalised Linear Mixed Effects Models (*GLMM*) with charr morph (Littoral or Pelagic) and month as predictors. Littoral and offshore habitat use was calculated as a percentage of the total number of positions. The sum of individual monthly density values for both offshore and littoral zones was used. Daily average values of fish displacement rates, depth and relative depth (fish distance from lake bed) were calculated from the tracking data as the mean of individual daily means. Individual fish identification was modelled as a random effect to account for observational dependency caused by repeated measures from the same individuals. Individual values of K50 and K95 estimates per month were used for analysing morph and season effects on home range. Track durations differed for individuals; periods of valid positional data are stated in Table S1. Gross patterns of fish activity were compared as average relative displacement between fixes, given in body lengths per second, BLs^−1^ to standardise for body length. Though this is a measure of speed, we describe it as displacement since activity is in all cases likely to be underestimated (since valid fish detections were approximately every 80 minutes). Displacement was calculated between consecutive positions of each fish. All statistical analyses were conducted in JMP v9.03 software (SAS institute Inc.) with a significance level *P α* = 0.05.

To explore littoral and offshore habitat selection by charr, monthly Jacobs index^42^ values (*D*) were calculated: *D* = (*r* − *p*)/(*r* + *p*  – *2rp*), where *r* is the proportion of habitat used and *p* is the proportion available*. D* was derived from all viable positions; and the 95% confidence limits of the individual monthly means were calculated to test whether they differed significantly from the ‘neutral’ value of 0, where habitat is used proportionally to its availability. If 0 was not included within the range of confidence limits, the use of habitat type was considered non-random and the habitat was either preferred (+*D*) or avoided (−*D*). This method was selected as only two habitats were defined (littoral and offshore), thus this index gives the full range of values (−1 < *D* < 1) for any value of *r* or *p*.

## Results

### Polymorphism and dietary niche within the study sample

**Figure 2:**
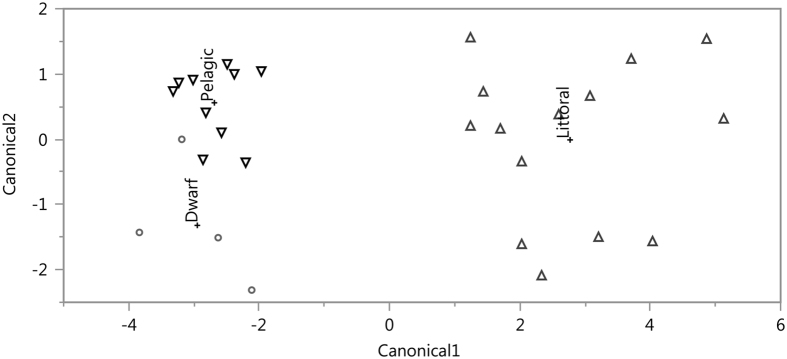
Ordination of the three discriminant functions (residuals derived from the regressions of Head length / Pelvic fin length, Head depth at eye /eye diameter and Head depth at operculum /eye diameter) selected according to a stepwise discriminant analysis model to test individual membership according to the three visually identified ecomorphs of Arctic charr from Lake Ellasjøen. Individuals are identified by markers according to their visually assigned morphology group; grey triangle, Littoral; black triangle, Pelagic; grey circle, Dwarf maturing. Group centroids are marked (+).

Morphometric data of 28 Ellasjøen Arctic charr (FL; 166–505 mm) were analysed (Table S1, Fig. 2). There was a highly significant relationship of each morphometric variable with FL (*R*^*2*^ = 0.65–0.97*, p* < 0.0001). The model selected the residual values of head length (HL)/Pelvic fin length (PEL), head depth at eye (HDE)/eye diameter (ED) and head depth at operculum (HDO)/ ED as the first (96.0% of variation), second (3.9% of variation) and third (0.1% of variation) discriminant functions respectively (Fig. 2). The canonical scores of the model revealed three well-separated groups (Dwarf-maturing and putative Littoral and Pelagic ecomorphs), with significant differences between the group centroids (Wilk’s Lambda = 0.09, *F* = 15.12, *df* = 6, *p* < 0.0001, Fig. 2). Fish were grouped according to the output of the discriminant model and checked for compatibility against existing biological and photographic data. In all cases these complied, except for one individual which, since it was a reproductively mature female (was expressing ova when abdomen gently stroked) at a FL of 23 cm, and showed classical parr markings was identified as a Dwarf Maturing charr (Fig. S2), even though the morphometric characteristics were similar to a Pelagic fish. These phenotype classifications were used to distinguish putative Littoral from putative Pelagic fish for comparison of spatial behaviour of these two groups.

**Figure 3:**
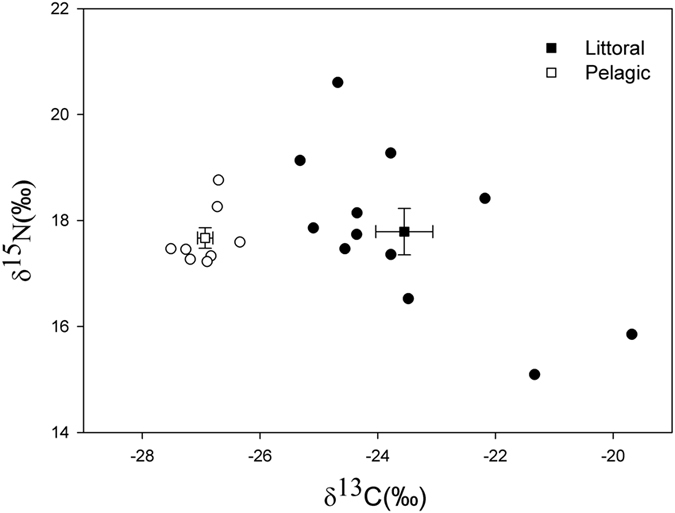
The individual values (circles) and mean values (squares) ± SE of δ ^15^N and δ^13^C in pelvic fin clips collected from Littoral (*n* = 12) and Pelagic (*n* = 8) Arctic charr sampled and tracked in Lake Ellasjøen from Lake Ellasjøen. A significant difference in δ^13^C between morphs was shown (*ANOVA*, 1 *df*, *F* = 35.86, *p* < 0.001).

Carbon and nitrogen stable isotope values of Pelagic charr varied between individuals from −27.5 to −26.3 (mean = −27.0) ‰ and from 16.1 to 18.8 (mean = 17.4) ‰ respectively. In Littoral charr values varied from −25.3 to −19.7 (mean = −23.5) ‰ and from 15.1 to 20.6 (mean = 17.8) ‰ for δ^13^C and δ ^15^N respectively. No significant difference was found in δ^15^N values between morphs, however a highly significant difference (*ANOVA*, 1 *df*, *F* = 35.86, *p* < 0.001) was observed in δ^13^C between morphs (Fig. 3, Table S2), with greater δ^13^C depletion evident for the Pelagic morph, consistent with greater zooplanktivory (Table S2).

### VPS deployment

The VPS was deployed for 360 days and generated 191,229 valid fish positions. From the sample of 24 tagged charr (14 Littoral [mean FL = 477 mm, range 308–505 mm; 10 Pelagic [mean FL = 267, range 212–314 mm]), no positions were recorded for the individual T17 (Pelagic), as this tag failed to transmit. Eighty six percent (12/14) and 67% (6/9) of Littoral and Pelagic morph tags respectively that provided data were still transmitting at the end of the study. The mean number of days for which valid track data were obtained for Littoral fish was 332 days and 256 days for Pelagic fish (Table S1). The number of positions derived per fish differed between months (*ANOVA*: *n* = 24, *F* = 15.91, 11 *df, p* < 0.0001) but not between morphs (*ANOVA*: *n* = 24, *F* = 0.23, 1 *df, p* > 0.05) (Table S3).

### Temporal patterns of activity and habitat use

**Figure 4:**
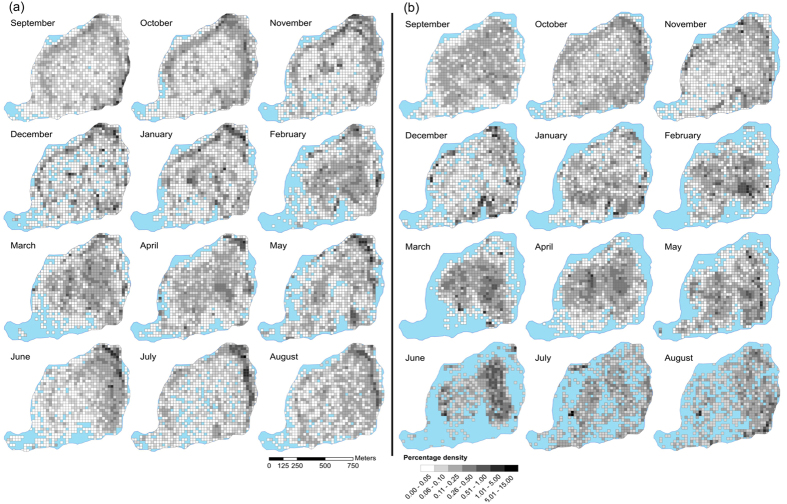
The percentage density distribution within Lake Ellasjøen of Littoral (a) and Pelagic (b) morph Arctic charr on a monthly basis, from September 2009 to August 2010. The number of fish positions and number of individual fish are stated per month in Table S3. Grid square area is 25 m^2^, percentage density increases with shading intensity, blue represents those lake areas for which no fish positions were recorded. Maps were created using ESRI (Environmental Systems Resource Institute http://www.esri.com/software/arcgis) ArcMap software, version 10.0, 2010. A high resolution of this figure is available as Supplementary Information.

**Figure 5:**
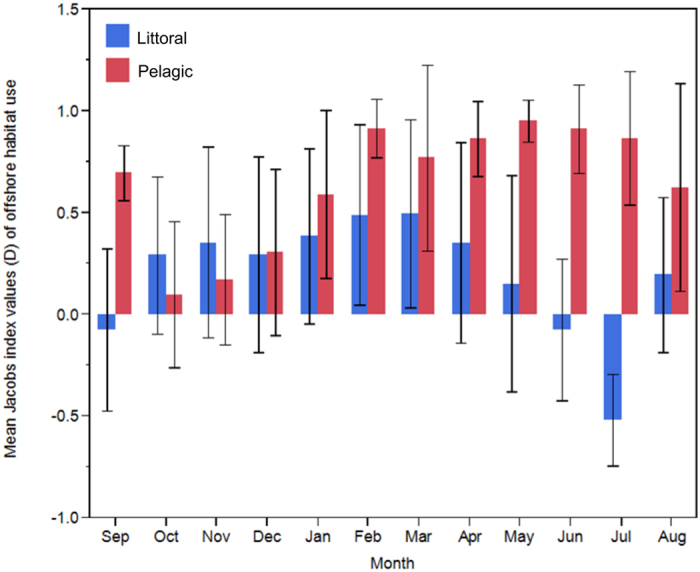
Monthly mean values of Jacobs selectivity index *D*, for the offshore habitat (lake depth 8–34 m, 77.4%) by Arctic charr in Lake Ellasjøen. *D* varies from −1 (strong avoidance) to +1 (strong preference), and values close to zero indicate that the habitat is used proportional to its availability. Error bars represent the 95% confidence limits of the means, if 0 was not included within the range of confidence limits, the use of the habitat type was considered not random but the habitat was either favoured or avoided (*p* < 0.05). Habitat use was calculated from tag position data, calculated as a percentage of total positions per morph per month. Months are listed from September 2009–August 2010.

Both morphs occupied the offshore zone more than the littoral zone (Table S3) with 73.09% of Littoral fish positions (Fig. 4a) and 92.42% of Pelagic fish positions (Fig. 4b) located in offshore habitat (mean of monthly mean values). Littoral morph charr utilised the littoral habitat, on average 19.3% more than Pelagic charr. The only month Pelagic fish occupied the littoral zone more than Littoral fish was in October (Fig. 4a,b). Both morphs showed variation in habitat use with month; greatest littoral use was recorded in July (53.96%) and October (21.62%) for Littoral and Pelagic morphs respectively (Fig. 4a,b). The littoral habitat was reduced during ice coverage (~16/12/2009–24/5/2010), with ice formation *ca*. 110 cm thick (Bjørnøya meteorological station, 2015, pers. comm.). Greatest use of offshore habitat was during February (83.02%) for Littoral charr and in June (98.90%) for Pelagic charr (Fig. 4a,b). Pelagic fish exhibited significant positive selection (high positive *D*) for offshore habitat in all months except October to December, when they exhibited no selection of either habitat (Fig. 5). Conversely, Littoral morph fish exhibited significant selection of littoral habitat in July and a non-significant preference in September and June (Fig. 5). Littoral morph fish mainly utilised the offshore habitat in the remaining months, however significant selection was only observed during February and March.

**Figure 6:**
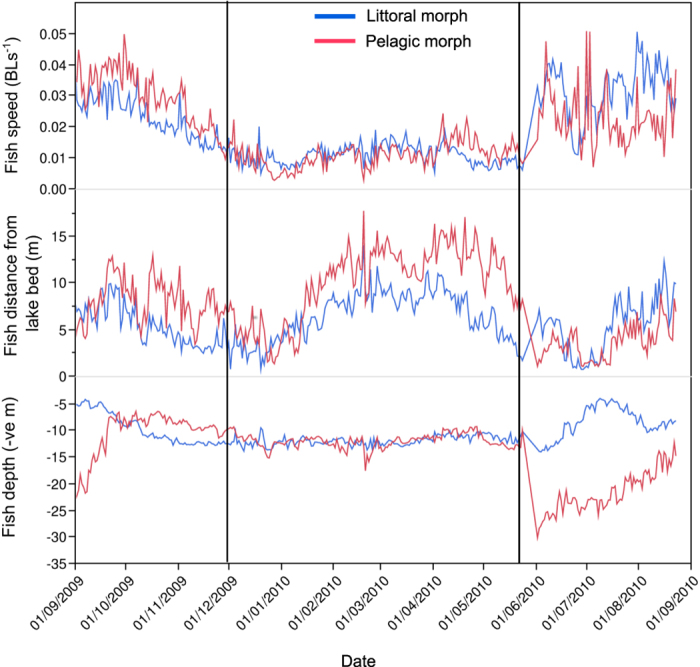
Daily average values of relative fish displacement (BLs^−1^), fish distance from lake bed (m) and fish depth (negative m) (*n* = 349), for the Littoral and Pelagic morphology groups of sampled Lake Ellasjøen Arctic charr. Average values were calculated as the mean of individual daily means per fish morphology. Estimated dates of lake ice formation (1/12/2009) and breakup (24/5/2010) are represented by the reference lines on the date axis.

A significant morph x month interaction (*F* = 4.61, 11 *df*, *p* < 0.0001) was observed for littoral habitat use. No significant morph x month interaction (11 *df*, *p* > 0.05) was found for offshore habitat use, but a significant effect of morph was observed (*F* = 26.14, 1 *df*, *p* < 0.0001) (Table 1, Fig. 4a,b). Significant morph x month interactions (*p* < 0.0001, 11 *df, n* = 7,165) were found for each of the response variables; fish displacement (*F* = 50.48), fish distance from lake bed (*F* = 21.54) and fish depth (*F* = 174.69) (Table 1, Fig. 6). Pelagic fish were significantly more active, further from the lake bed and at greater depth than Littoral morph fish. Mean values (calculated as mean of monthly means) of fish displacement, distance from lake bed and depth were; 0.069 BLs^−1^, 8.21 m and 14. 11 m for Pelagic fish and 0.047 BLs^−1^, 5.87 m and 10.47 m for Littoral fish (Fig. 6, Table S3).

**Figure 7:**
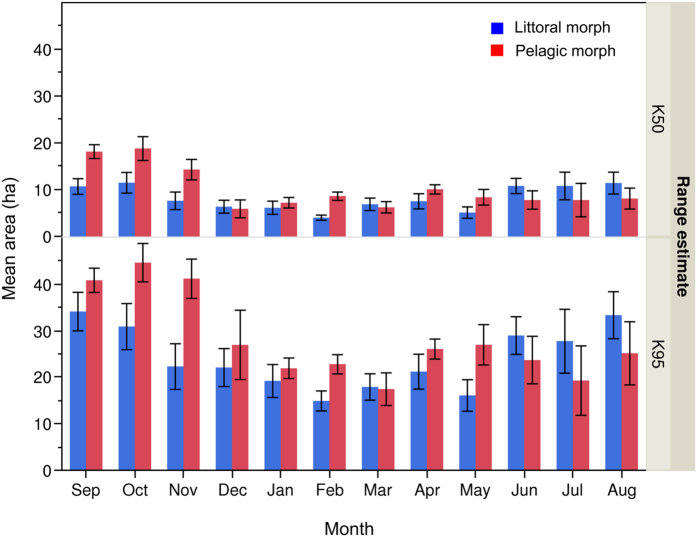
Mean monthly home range areas (hectares) of Littoral and Pelagic morph Arctic charr, calculated using the kernel analysis estimates; K50 (top) and K95 (bottom) that have been clipped to within the boundary of Lake Ellasjøen, Bear Island. Bars are coloured according to the charr morph, *n* of individuals varies for each month; values are stated in Table S3, total *n* = 255. Months are listed from September 2009–August 2010. Error bars represent +/− one standard error.

Ninety five percentile kernel (K95) home range differed between months (*F* = 5.51, *p* < 0.0001, 11 *df*), but not between morphs (*F* = 1.77, *p* > 0.05). Significant morph x month interaction occurred for K50 estimates of home range area (*F* = 2.11, *p* = 0.0202, 11 *df*) (Table 1). K50 area of Pelagic charr, averaged over the year, was 25.6% greater than for Littoral charr (Fig. 7, Table S3). Mean home range was consistently greater for Pelagic fish through the autumn months (Fig. 7).

## Discussion

This is the first study to derive fine-scale habitat and behaviour patterns from sympatric population components over their complete and natural habitat, over an annual timescale. Mallet *et al.*^43^ emphasize the importance of behavioural ecological insights with regard to understanding evolutionary processes in sympatry, and although studies of sympatric ecomorphs in post-glacial lakes have sampled morphs in different habitats and interpreted niche use from integrated diet and parasite fauna^10^,^19^,^27^,^44^,^45^ the fine-scale space-use dimension has been missing. While we found clear differences in the spatio-temporal distribution and behaviour of the two sympatric Arctic charr morphs we tracked, space use overlapped substantially, such that spatial segregation linked to trophic polymorphism, was incomplete and dynamic. Fine-scale patterns of habitat use and activity changed with season for both morphs, but the temporal patterns differed between the phenotypes. Over an annual timescale these space-use differences were correlated with long-term diets, defined by δC^13^ values, and concordant with pelagic (Pelagic morph) versus generalist benthic (Littoral morph) lifestyles.

Several studies have shown the importance of foraging specialisations in the evolution of population structure, for example in killer whales (*Orcinus orca*)^7^, while Harrod *et al.*’s^19^ and Præbel *et al.*’s^10^ whitefish (*Coregonus lavaretus*) experiments, have found long-term niche overlap in sympatric morphs to be low in terms of macro-habitat and diet. Based upon outcomes of stable isotope studies, it is hypothesised that intraspecific niche separation lowers resource competition^46^. The significant difference in δ^13^C values between the two charr morphs in our study reflects long-term partitioning in feeding habitat, with the lower values of δ^13^C in Littoral morph charr reflecting a strong reliance on littoral, epibenthic energy sources, when compared to the zooplankton-feeding Pelagic morph fish^32^ (Table S2 provides stable isotope data for key food web components from Ellasjøen). As both morphs occupied the same trophic level (no significant difference was observed in δ^15^N between Littoral and Pelagic morphs) niche separation between the morphs is unlikely to be a predator-prey response. The unusually high δ^15^N levels in Ellasjøen charr result from the high input to the system by marine birds at a relatively high trophic level^47^. In instances of interspecific competition, habitat niche shifts are commonly observed in Arctic charr e.g.^28^,^48^,^49^. However in Ellasjøen, where Arctic charr is the only fish present, intraspecific resource competition is the likely driver of the polymorphism identified, serving to facilitate specialisation on subsets of the range of habitat and food resources available in this low-productivity, high-latitude aquatic environment.

Divergence of phenotypes along the pelagic - littoral habitat axis is the most common form of resource-driven polymorphism observed in postglacial lakes^50^. In addition to divergence into pelagic and benthic niches, further divergence into more specialised niches has also been documented e.g. into profundal^45^, prey specific benthic^44^ or piscivory niches^21^. The discrete Littoral and Pelagic morphs found in Ellasjøen are separated in both morphological traits and niche use, which is concordant with habitat selection (littoral or pelagic) and long-term diet (defined by δC^13^ values). The Dwarf-maturing and Other charr forms identified during processing but not tracked during this study are likely an epibenthic feeding ‘dwarf’ phenotype e.g.^24^ and an ontogenetic juvenile grouping. Therefore the Ellasjøen charr population likely comprises three sympatric ecomorphs; a littoral epibenthic (Littoral) morph, an offshore planktivorous (Pelagic) morph and a profundal epibenthic (Dwarf maturing) morph. Examples of such polymorphism have been repeatedly shown in post-glacial aquatic systems as these ecosystems offer several discrete foraging resources for fish, which may serve to reduce competition^16^,^19^, with population structure often more complex with increasing lake size^51^,^52^.

The Littoral fish were exhibiting the red/orange spawning colouration, typical of Arctic charr, and exhibited secondary sexual characteristics at the time of capture (late August) and the Dwarf-maturing fish were releasing gametes, consequently spawning of these two morphs likely occurred soon after capture. Pelagic fish did not exhibit indicators of approaching spawning, however most often, but not always (e.g. Windermere, England^8^), Arctic charr spawn in the autumn so that fry emergence coincides with plentiful food in the following spring. It is therefore probable that Elasjøen Pelagic fish spawn in late autumn, as observed in Thingvallavatn, where four charr phenotypes segregate with respect to both spawning time and habitat^24^. In Ellasjøen, the only months in which Pelagic charr utilised the littoral zone more than Robust were during October and November, where this morph occupied significantly shallower depths than during other months. It is therefore possible that these fish were utilising the littoral region for spawning in this period. Alternatively, they could have used deep-water gravel substrate as found for Windermere, spring-spawning charr^8^. The occurrence of allochrony (temporal spawning variation), with respect to reproductive isolation between Ellasjøen morphotypes has not been investigated; neither has genetic analysis been undertaken. Without this knowledge, it is not possible to further infer the degree of ecological divergence within the population.

Despite long-term resource partitioning, short-term patterns of spatial distribution largely defined the behavioural differences between the Ellasjøen morphs. This was most distinct directly after ice break, in late May, when Pelagic fish moved into deeper water, close to the lake bed and Littoral fish were at shallow depths, exclusively within the littoral zone. This is likely a seasonal response to food availability, corresponding to a peak in feeding by the Littoral morph on littoral prey resources i.e. young-of-year charr and zoobenthos and predominant use, by the Pelagic morph, of zooplankton in the water column and emerging chironomids^31^. Pelagic charr often occupied larger home range areas and were usually more active than Littoral charr, consistent with a more pelagic, planktivorous mode of foraging^27^,^53^. Only during summer was Littoral charr activity (both displacement and home range area) greater than that of the Pelagic morph. This pattern of intense activity by the Littoral morph, coupled with a reduction of metabolism in a period of reduced rations (i.e. winter), has been shown in ectothermic animals^54^ and may account for the annual variation in activity for this morph, supporting an energy conservation strategy in periods of resource scarcity. During winter ice-cover average fish displacement (for both morphs) was reduced by over 50% when compared to ice-free conditions, and home range area was significantly smaller. However, Pelagic fish remained more active and occupied more open water i.e. they were further from the lake bed and further from the lake edge than Littoral fish, although Littoral fish migrated into deeper water during this period, possibly due to habitat restriction due to thick ice at the lake edge, or to occupy warmer water temperatures. Hayden *et al.*^30^ have shown that Arctic fish can alter their trophic ecology from being summer planktivores to winter benthivores in order to exploit this natural variation in prey abundance. It is possible this strategy was employed by the Pelagic morph charr in our study. However, despite synchrony with the annual fluctuations in resource availability, it is likely that resource competition is intensified in winter, resulting from depletion of resources and restriction of littoral habitat^55^. The relative contribution of littoral and pelagic production for whole ecosystem metabolism can be highly spatially and temporally variable within a lake and Arctic charr have evidently adapted to seasonal fluctuations in food availability and composition. Such generalist foraging by fish has been shown to be particularly evident in the high-latitudes where consumers must adapt to dramatic seasonal changes in prey availability, light and temperature^29^,^30^. Understanding the role of spatio-temporal aspects of differentiation in ecological niche use among model-organism population components, in this case of charr, could be strengthened with improved knowledge of the physiological processes by which they may occupy different niches. The physiological requirements and feeding behaviour of fish exhibiting dietary specialisation is highly dependent on individual morphology because this affects foraging efficiency, predation risk, competitive dominance and basic metabolic rate. As Littoral and Pelagic morphs exploit environments with different feeding opportunities, temperature and light conditions, it might be expected that variation in aerobic metabolism and growth efficiencies in relation to water temperature would be observed, which to our knowledge has not been investigated.

Changes in temperature, snow, ice-cover, and nutrient availability exert major influences on the biological dynamics in the Arctic, and extensive ecological consequences of recent warming-related trends in these parameters will affect the ecology of Arctic lakes^56^. Understanding temporal patterns of habitat and resource use is necessary to predict potential effects of, for example species invasions on the functioning of high-latitude aquatic ecosystems which have low biodiversity and are considered particularly susceptible to environmental change^57^. Hence recognising the factors determining the resource use of Arctic charr, the most northerly distributed freshwater fish, is fundamental for evaluating the possible impacts of various disturbances on these ecosystems, including climate induced shifts in species composition^58^,^59^ and the interactions between phenotypes adapted to specific niches^60^.

Improved understanding of the mechanisms involved in habitat segregation and niche differentiation within and between populations requires an integrated approach to measuring individual responses along physiological, ecological and behavioural axes in response to differing conditions. Among freshwater fishes, great progress has been made using the threespined stickleback (*Gasterosteus aculeatus*) model, employing population genomic methods^29^,^61^. But for incorporating behavioural ecology insights in the natural environment, larger animals such as charr and whitefish offer major opportunities for combining detailed individual behaviour data *in situ* by telemetry, alongside genetic, morphometric and dietary data. For Ellasjøen charr there is a need to determine whether there are population genetic differences between the morphs and if so, whether reproductive segregation occurs and by what means. A wide range of studies combining common garden experiments as well as population genetic and resource use studies of ecomorphs have demonstrated a tendency for Arctic charr ecomorphs to exhibit population genetic differences and a degree of reproductive isolation^22^,^23^,^24^, while also maintaining strong epigenetic effects^5^. However, a limited number of well-studied charr systems exist, and it seems likely that these can be relatively labile systems. Winifred Frost and subsequent workers through the 1950s to the 1980s (see^8^ for review) found that Windermere charr exhibited two morphs, putatively reproductively isolated by spawning season (spring and autumn) and location, each with different numbers and length of gill rakers, and which fed largely on zooplankton. But Corrigan *et al.*^25^,^49^ found that by ~2005, spring and autumn spawning Windermere charr no longer exhibited any difference in gill raker number or gill raker length, had switched diet towards generalist benthivory, were only partially reproductively isolated (determined by population genetic methods) and that population genetic differentiation was decoupled from phenotypic differences. Thus, further insight into the mechanisms by which phenotypic and genetic divergence may occur between, or be lost from, sympatric subpopulations is needed.

The potential combination of individual genetic screening and morphometrics, combined with temporal evaluation of spatial behaviour and diet in lake habitats with differing niche opportunities, provides strong opportunities for dissecting the contributions of environment and genotype on evolutionary divergence of phenotype or retention of phenotypic plasticity. The majority of studies addressing ecological questions do so under relatively controlled conditions, either by experimental regulation of the environment or conducting field studies of a limited duration. Such approaches, while vital to providing experimental evidence of plausible mechanisms, may fail to address the underlying variability inherent in almost all ecosystems. Hendry^17^ proposes that future study of population divergence should identify different populations across the speciation continuum and then investigate how various factors, influence progress along the continuum, as well as transitions between the states. Ellasjøen Artic charr present a relevant case for further study; they are polymorphic, shown by longer-term patterns of divergence in phenotype, behaviour and resource use, yet fine-scale patterns reveal periods of spatial overlap. It has not yet been determined to what degree reproductive isolation occurs between these phenotypes, this would help define the current state of population divergence as well as provide further insight into the factors that influence progress both toward or against the development of speciation.

## Additional Information

**How to cite this article**: Hawley, K. L. *et al.* Fine-scale behavioural differences distinguish resource use by ecomorphs in a closed ecosystem. *Sci. Rep.* **6**, 24369; doi: 10.1038/srep24369 (2016).

## Supplementary Material

Supplementary Information

Supplementary Image

## Figures and Tables

**Table 1:** Results of *GLMM* analysis of behaviour and habitat use variance with charr morph (Littoral or Pelagic) and month as predictors.

	Morph (1 df)			Month (11 df)		Morph x Month (11 df)	
	*n*	*F*	*p*	*F*	*p*	*F*	*p*
Littoral habitat use (%)	255	3.03	0.0963	3.03	0.0009	4.61	<0.0001
Offshore habitat use (%)	255	26.14	<0.0001	1.36	0.1919	1.42	0.1664
Fish displacement (BLs^−1^)	7165	7.41	0.0127	280.34	<0.0001	50.48	<0.0001
Fish distance from lake bed (m)	7165	2.54	0.1261	117.97	<0.0001	21.54	<0.0001
Fish depth (m)	7165	2.60	0.1217	81.68	<0.0001	174.69	<0.0001
K50 (ha)	255	2.55	0.1248	6.91	<0.0001	2.11	0.0202
K95 (ha)	255	1.77	0.1971	5.51	<0.0001	1.52	0.0661

Individual fish identification was modelled as a random effect. Littoral and offshore habitat use was calculated as a percentage of the total number of viable positions within each zone per month per morph group. The sum of individual monthly density values for both offshore and littoral zones was used. The response variables; fish displacement (BLs^−1^), fish distance from lake bed (m) and fish depth (m) were derived from the tracking data, individual daily mean values were used. Home range area was estimated from a random sample of 54 positions per individual per month. *GLMM* was calculated from the individual estimates of K50 and K95 per month.
