# Supplementary material for: Fine-scale behavioural differences distinguish resource use by ecomorphs in a closed ecosystem
Source: Sci Rep. 2016 Apr 21;6:24369. doi: 10.1038/srep24369 (PMC4838883; doi:10.1038/srep24369)

Appendix S1: Electronic Supplementary Information

Fine-scale behavioural differences distinguish resource use by ecomorphs in a closed ecosystem

Kate L. Hawley[*][a, b], Carolyn M. Rosten[c], Guttorm Christensen[d] and Martyn C. Lucas[b]

[a] Norwegian Institute for Water Research (NIVA), Gaustadalléen 21, 0349 Oslo, Norway

[b] School of Biological and Biomedical Sciences, Durham University, Science Laboratories, South Road, Durham, DH1 3LE, UK

[c] Norwegian Institute for Nature Research (NINA), Høgskoleringen 9, 7034 Trondheim, Norway

[d] Akvaplan-NIVA, Fram Centre, 9296 Tromsø, Norway

[*] Corresponding author: kate.hawley@niva.no

Materials and methods

FISH SAMPLE PROCESSING PROCEDURE

Fish were individually anesthetised in a benzocaine solution (0.5 ml l^{-1}). Once sedated the fish were placed on a latex mat, which was sterilised (wiped with ethanol, and allowed to dry) between each fish. A 2 cm incision was then cut through the ventral body wall and peritoneum of the fish using a scalpel. A pre-sterilised (washed in 96 % ethanol and dried) tag was inserted into the incision, rounded end first, and positioned to sit horizontal in the abdominal cavity. The incision was then closed using two monofilament sutures. The entire procedure was conducted in as sterile conditions as possible. Scalpel blades and suture needles were changed between each fish and latex gloves were worn by the surgeon. Each fish was then allowed to recover in a large tank, until full reflexes and movement had returned, and no visible impairment as a result of surgery observed. The fish were then returned to the lake, as it was considered that a swift release provided the fish with the least stressful from of recovery.

Thirty two charr, including the 24 fish that were subsequently tagged, were sedated and a series of morphometric measurements were taken (Table S1). Head measurements, including; head length (distance from the tip of the snout to the operculum), head depth at operculum, head depth at eye, lower jaw length and eye diameter were selected based on their relationship to prey acquisition and handling. Body measurements, including; fork length, caudal peduncle depth, pelvic and pectoral fin lengths were also used to describe individual body shape. Body shape morphology is generally adapted to different modes of swimming in different foraging habitats. All measurements were taken from the left side of the fish, using precision callipers and measured to the nearest 0.1 mm.

Table S1: Summary of the sampling protocol undertaken for each tagged Arctic charr, sampled from Lake Ellasjøen. Summary of the period of VPS derived individual fish positions is also stated, as determined by temporal tracking data. The date of final position is stated, as well as the number of days of data, where total study duration was 357 days.

Fish ID	Fork length (mm)	SIA	Photo	Morph group	Date of final position	Total n of position days
T01	405	Y	Y	Littoral	23/08/2010	349
T02	435	Y	Y	Littoral	16/07/2010	310
T03	495	Y	Y	Littoral	23/08/2010	344
T04	505	N	Y	Littoral	09/06/2010	247
T05	418	Y	Y	Littoral	23/08/2010	357
T06	297	Y	Y	Pelagic	19/06/2010	283
T07	362	Y	Y	Littoral	23/08/2010	339
T08	212	Y	Y	Pelagic	25/01/2010	133
T09	420	Y	Y	Littoral	23/08/2010	347
T10	314	Y	Y	Pelagic	23/08/2010	347
T11	460	Y	Y	Littoral	23/08/2010	349
T12	240	N	N	Pelagic	23/08/2010	323
T13	385	Y	Y	Littoral	23/08/2010	338
T14	350	Y	Y	Littoral	23/08/2010	344
T15	398	Y	Y	Littoral	23/08/2010	346
T16	275	Y	Y	Pelagic	23/08/2010	294
T17	265	N	Y	Pelagic	Tag failed to transmit	0
T18	392	Y	Y	Littoral	23/08/2010	344
T19	281	Y	Y	Pelagic	23/08/2010	336
T20	224	Y	N	Pelagic	23/08/2010	287
T21	308	N	Y	Littoral	23/08/2010	349
T22	290	Y	Y	Pelagic	23/08/2010	286
T23	271	Y	Y	Pelagic	07/06/2010	268
T24	369	Y	Y	Littoral	23/06/2010	283
T25	166	Y	Y	Dwarf	Not tagged	-
T26	248	Y	N	Dwarf	Not tagged	-
T27	251	Y	Y	Other	Not tagged	-
T28	219	Y	Y	Dwarf	Not tagged	-
T29	175	Y	Y	Other	Not tagged	-
T30	178	N	Y	Other	Not tagged	-
T31	220	N	Y	Dwarf	Not tagged	-

Table S2: The mean and standard deviation of $\delta^{15}N$ and $\delta^{13}C$ in pelvic fin clips collected from Littoral (n = 12) and Pelagic (n = 8) Arctic charr sampled from Lake Ellasjøen, Bear Island. * indicates a significant difference in $\delta^{13}C$ between morphs (ANOVA, 1 *df*, F=35.86, p <0.001). [1] indicates G. Christensen unpublished data of $\delta^{15}N$ and $\delta^{13}C$ for zooplankton and chironomid samples collected from Ellasjøen, July 1996.

Sample type	n	$\delta^{15}N$ ‰	S.D.	$\delta^{13}C$ ‰	S.D.
Littoral sample	12	17.57	1.45	-23.56*	1.61
Pelagic sample	8	17.67	0.51	-26.93*	0.35
Zooplankton[1]	1	11.3		-33.6	
Chironomids[1]	9	12.6	0.8	-27.7	2.2

Table S3: Summary information of telemetry derived data for Littoral (L) and Pelagic (P) morphs of Arctic charr in Lake Ellasjøen, Bear Island. The percentage of fish positions located in either the littoral (lake depth 0 – 8 m) or offshore habitat (8 – 34 m) are presented. Least squares mean values of fish displacement (BLs^{-1}), fish distance from lake bed (m) and fish depth (m) are shown as well as K50 and K95 home range estimates (ha). Total means were calculated as the mean of monthly means per morphology group, standard error (S.E) is shown.

	n individuals		n positions		% fish positions littoral zone		% fish positions offshore zone		Fish displacement (BLs^{-1})		Fish distance from lake bed (m)		Fish depth (m)		K50 (ha)		K95 (ha)	
Month	L	P	L	P	L	P	L	P	L	P	L	P	L	P	L	P	L	P
Sep	14	9	11704	7279	34.74	5.34	65.26	94.66	0.072	0.137	7.60	8.57	6.04	13.80	10.66	18.12	34.11	40.82
Oct	14	9	12188	8042	21.40	21.62	78.60	78.38	0.057	0.120	5.28	8.59	10.54	7.85	11.46	18.78	30.88	44.62
Nov	14	9	11293	7374	25.05	18.74	74.95	81.26	0.040	0.079	3.44	7.10	12.50	9.52	7.59	14.27	22.29	41.17
Dec	14	9	7807	5027	26.41	14.98	73.59	85.02	0.024	0.036	2.91	4.33	12.50	11.73	6.34	5.88	22.07	26.95
Jan	14	9	11780	7301	21.17	9.12	78.83	90.88	0.024	0.029	5.66	7.16	12.16	12.06	6.12	7.19	19.19	21.92
Feb	14	8	7825	4739	16.98	1.35	83.02	98.65	0.031	0.037	8.54	12.10	12.32	13.04	4.00	8.95	14.90	23.15
Mar	14	8	11591	6756	17.89	6.04	82.11	93.96	0.034	0.041	8.67	12.31	12.31	12.11	6.86	6.58	17.90	17.81
Apr	14	8	11007	6664	22.30	2.15	77.70	97.85	0.028	0.056	7.98	13.96	11.03	10.44	7.49	10.41	21.19	26.42
May	14	8	7850	4987	30.15	0.66	69.85	99.34	0.020	0.043	4.38	10.14	11.30	13.82	5.08	8.71	16.08	27.35
Jun	14	5	7965	1818	32.91	1.10	67.09	98.90	0.072	0.087	4.13	4.02	11.13	25.22	10.77	8.28	28.94	24.47
Jul	12	5	5634	1515	53.98	3.50	46.02	96.50	0.075	0.073	4.66	4.90	5.08	21.87	10.71	7.96	27.67	19.31
Aug	11	5	4891	1374	19.96	6.40	80.04	93.60	0.087	0.085	7.25	5.29	8.72	17.84	11.14	8.27	32.85	25.17
mean	14	8	9295	5240	26.91	7.58	73.09	92.42	0.047	0.069	5.87	8.21	10.47	14.11	8.18	10.28	24.00	28.26
S.E.			746	707	2.96	2.06	2.96	2.06	0.007	0.010	0.59	0.97	0.73	1.47	0.76	1.26	1.92	2.58

Figure S1: Mean daily water temperature (°C) at 3, 25 and metre depths in Lake Ellasjøen, Bear Island, over the study period (28/8/2009 – 23/8/2010). The duration of daylight, as hours between daily dawn and dusk is shown. Dashed reference lines on the date axis show the period of inverse temperature gradient (16/12/2009 – 24/5/2010, 158 days), inferring the likely period of complete ice coverage.

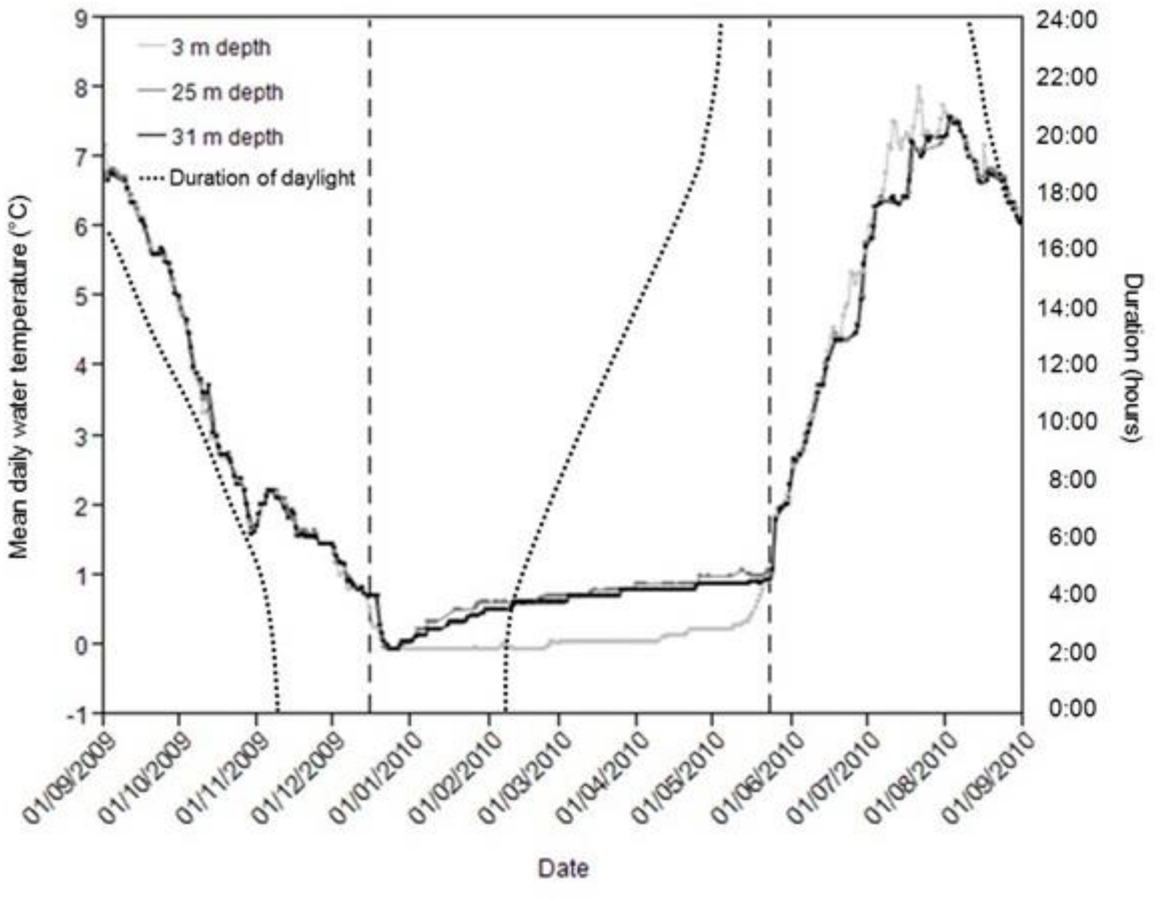

Figure S2: An example individual from each of the four visually determined groups, from a sample of 32 Arctic charr from Lake Ellasjøen. The groups were derived according to size, body shape, maturity, colouration and markings. The charr classified as Other (n =3) were not investigated further in this study as this group are a juvenile, intermediate form.

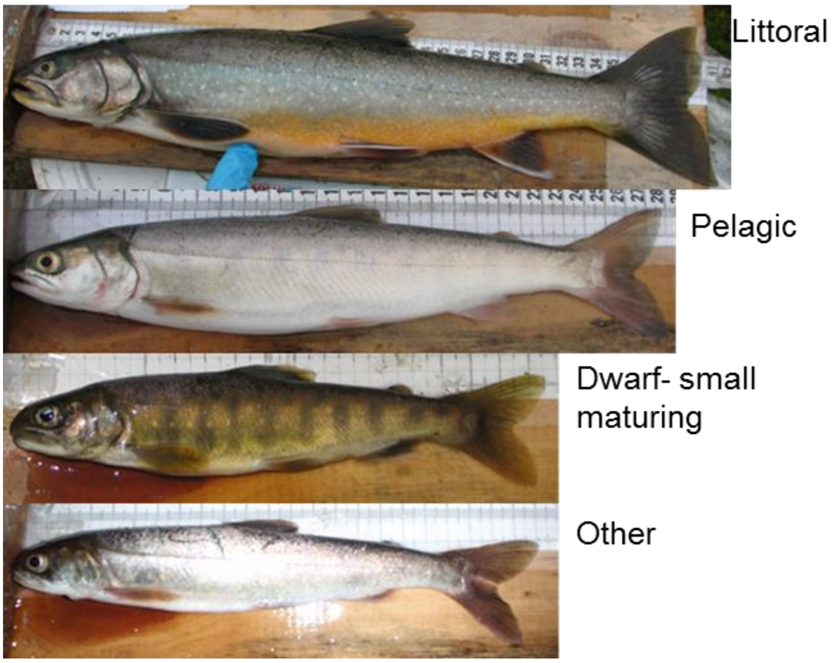

Supplement: Supplementary Information [file srep24369-s1.pdf]
