# Supplementary figures and images for: Fine-scale behavioural differences distinguish resource use by ecomorphs in a closed ecosystem
Source: Sci Rep. 2016 Apr 21;6:24369. doi: 10.1038/srep24369 (PMC4838883; doi:10.1038/srep24369)

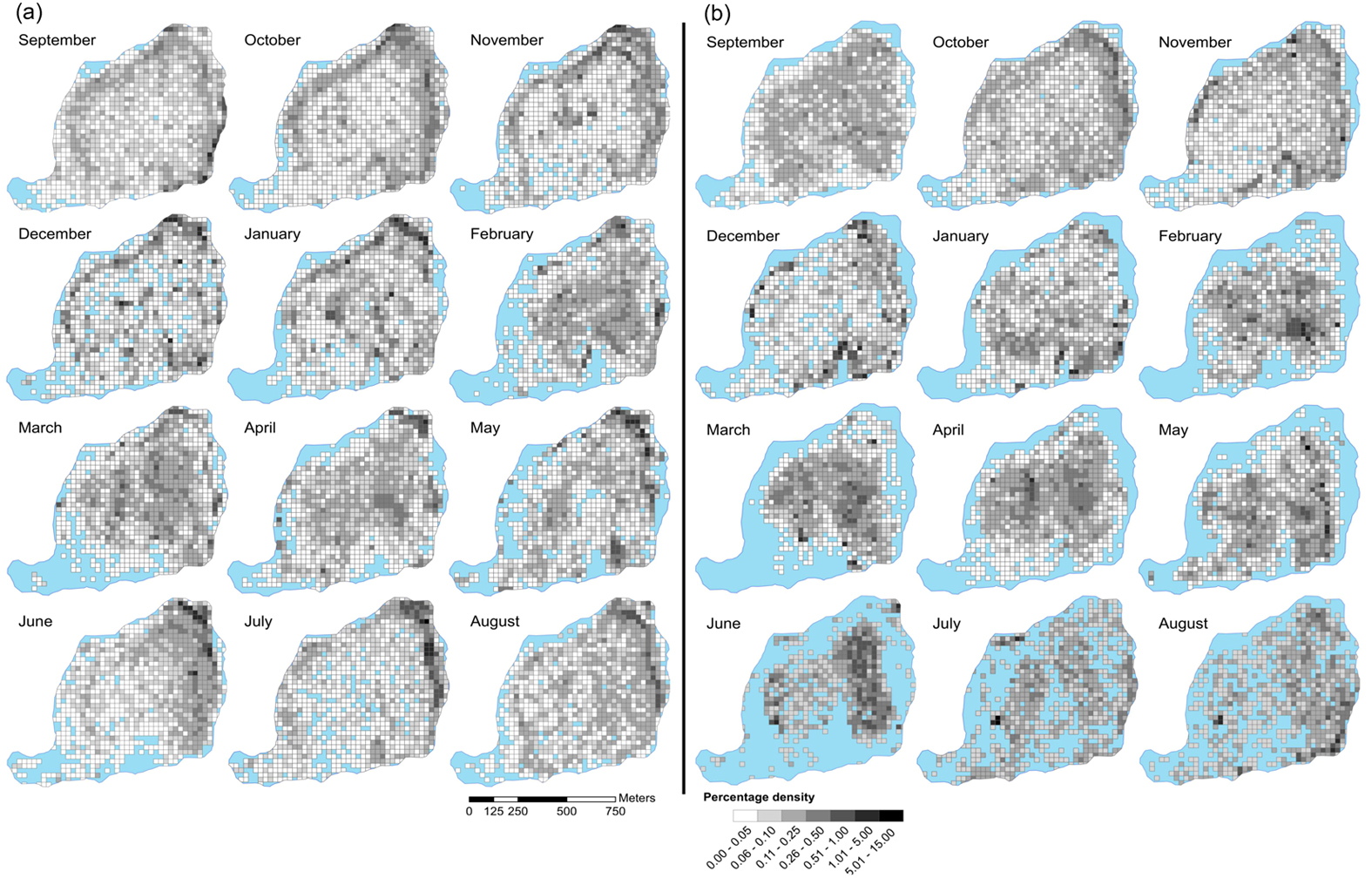

Supplement: Supplementary Image [file srep24369-s2.jpg]
